# Mediterranean diet and spirituality/religion: eating with meaning

**DOI:** 10.1007/s40520-024-02873-w

**Published:** 2024-11-19

**Authors:** Ligia J. Dominguez, Nicola Veronese, Francesco Saverio Ragusa, Valentina Petralia, Stefano Ciriminna, Giovanna Di Bella, Piero Schirò, Shaun Sabico, Nasser M. Al-Daghri, Mario Barbagallo

**Affiliations:** 1 Department of Medicine and Surgery, Kore University of Enna, Piazza Dell’Università, 94100 Enna, Italy; 2 Department of Health Promotion, Mother and Child Care, Internal Medicine and Medical Specialties, University of Palermo, Palermo, Italy; 3 Primary Care Department, Provincial Health Authority (ASP) of Palermo, Palermo, Italy; 4 Chair for Biomarkers of Chronic Diseases, Biochemistry Department, College of Science, King Saud University, 11451 Riyadh, Saudi Arabia

**Keywords:** Mediterranean diet, Spirituality, Religion, Conscious eating, Western diet

## Abstract

The interest in the Mediterranean diet has grown considerably due to its potential health benefits on the prevention of diverse age-related chronic diseases and its association with longevity. This dietary pattern, considered among the healthiest in the world, is not simply a combination of healthy foods but goes further in its historical and cultural roots. Mediterranean diet is not intrinsically tied to any specific religion or spiritual system, but its cultural and geographical context has influenced the dietary practices of its inhabitants, encompassing the history of Western civilization and of the three Monotheistic religions Christianity, Judaism, and Islam. These religions may have some impact on dietary choices due to religious customs and practices. In 2010 the Mediterranean diet was inscribed on the UNESCO’s Representative List of Intangible Cultural Heritage of Humanity, highlighting it as a social and cultural expression of the different food cultures of the Mediterranean region and indicating that the importance of this dietary and lifestyle pattern lies not only in its specific foods and nutrients, but in the way in which its characteristic foods are produced, cooked, and eaten. In this narrative review we will discuss the possible connections between the main religions originated in the Mediterranean basin and their influence on the composition of the Mediterranean diet, and the links between spirituality/religion and this dietary pattern. This traditional model can represent a form of conscious healthy eating and lifestyle in contrast to the unhealthy Western lifestyle and ultra-processed food consumption widespread throughout the world.

## Introduction

**Fig. 1:**
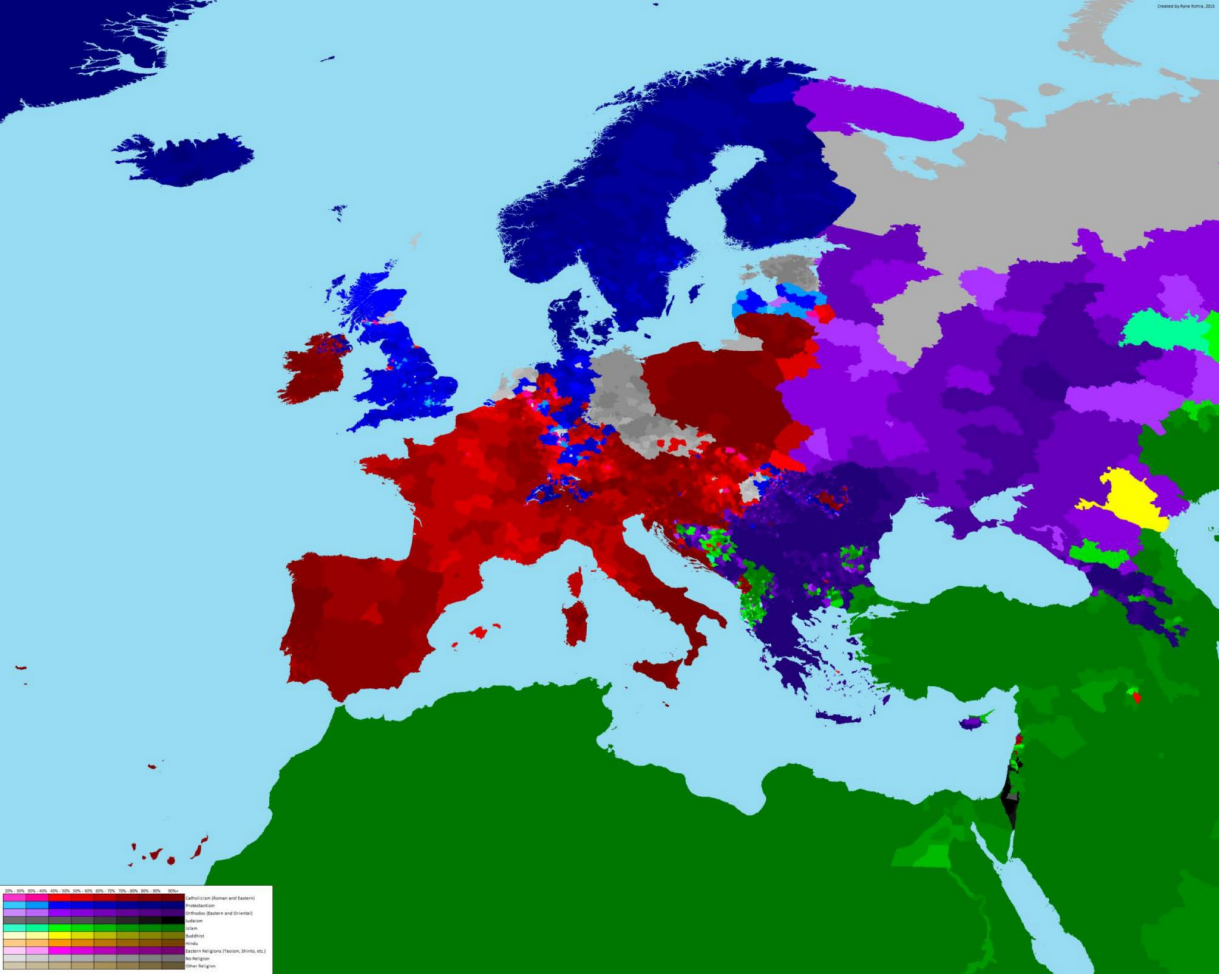
A detailed map of the distributions of religions in the Mediterranean basin and European countries (https://images.app.goo.gl/3EehadrpZqwQjcvF6 accessed 18/09/2024)

The Mediterranean diet, a dietary pattern inspired by the traditional eating habits of people living in the Mediterranean region, is considered today among the healthiest diets, with evidence of association with increased longevity and prevention of many non-communicable diseases, including cardiovascular disease, diabetes, dementia and some types of cancer [1]. This dietary pattern is not inherently tied to any specific religion or spiritual system, but the cultural and geographical context of the Mediterranean basin has influenced the dietary practices of its inhabitants. Its geographic and evolutionary origins are of much interest as they encompass the history of Western civilization. Religions such as Christianity, Judaism, and Islam had their beginning and are prevalent in the Mediterranean area (Fig. 1), and they may have some impact on dietary choices due to religious customs and practices [2]. In fact, inhabitants of the Mediterranean basin, as it also happens with other traditional ways of eating throw-out the world, consider food as an integral part of family celebrations, special days of honor, and festivals.

**Fig. 2:**
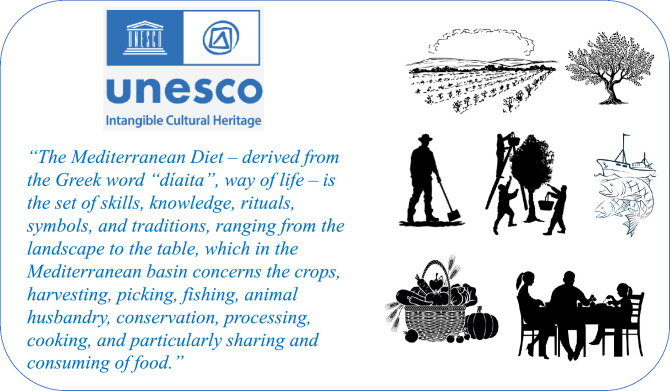
Official acknowledgment of the Mediterranean diet by UNESCO that was inscribed into the List of Intangible Cultural Heritage of Humanity

The relationship between spirituality and the Mediterranean diet is more subjective and personal, as spirituality can involve a range of beliefs and practices that may or may not be directly tied to a structured religion or to specific dietary choices. However, some individuals may find connections between their spiritual or philosophical beliefs and the principles of the Mediterranean diet as will be discussed. In this context, it is essential to consider that the Mediterranean diet is not simply a mere combination of healthy foods but goes further in its historical and cultural roots. So much so, that on November 16, 2010, the Mediterranean diet was inscribed on the United Nations Educational, Scientific and Cultural Organization (UNESCO) Representative List of Intangible Cultural Heritage of Humanity [3]. The Mediterranean diet is a historical, social, cultural, territorial, and environmental heritage, which has been transmitted from generation to generation for centuries, and it is closely connected with the lifestyles of the Mediterranean people throughout their history [4]. The recognition by UNESCO (Fig. 2) [5] highlights it as a social and cultural expression of the different food cultures of the Mediterranean basin and emphasizes the notion that the importance of this dietary and lifestyle pattern is not just in its specific foods and nutrients, but in the way in which its characteristic foods are produced, cooked, and eaten. Indeed, besides its food composition (vegetables, fruits, olive oil, whole cereals, legumes, nuts, fish, low amounts of meat and meat products, and wine in moderation if not contradictory to religious and social norms or health conditions), the Mediterranean diet includes various other components not directly related to its nutrient composition [1] (Table 1).

**Table 1:** Components of the Mediterranean diet not directly related to its nutrient composition

• Connection and respect for nature
• Moderate portions
• Moderate physical activity daily
• Prepare and consume meals in the company of other people (e.g., family and friends)
• Have adequate rest (sufficient and quality sleep at night; possibly short periods of rest during the day (“siesta” = nap)
• Preference for fresh, locally produced foods that have minimal industrial processes
• Cuisine pleasant to the palate

In this narrative review we will discuss the possible connections between the main religions that originated in the Mediterranean basin and their historical influence on the composition of the Mediterranean dietary pattern, as well as the possible links between spirituality/religion and the Mediterranean diet. This traditional culture can represent a form of conscious healthy eating and lifestyle in contrast to the usual unhealthy Western lifestyle and food consumption so widespread today throughout the world.

## Brief history of the Mediterranean diet (starting long before Ancel Keys)

Even if the notion of the Mediterranean diet as it is conceived today in nutritional research was first introduced by Ancel Keys who led the Seven Countries Study in the 1950s [6], historical, traditional, and literary sources confirm that the traditional Mediterranean eating habits and lifestyle were originated centuries before, and remained similar in the same geographical areas [1]. The Mediterranean dietary model comprises food choices and lifestyle along with traditions, historical knowledge, abilities, and practices, which have been transmitted over time through generations, including elements such as the territory, cultivations, crop production and management, harvesting and cooking, providing a sense of belonging and permanency to the community. Mediterranean traditional gastronomies are rich in flavors, colors, aromas, and memories, emphasizing the taste and harmony with nature, while giving significance of preparing and consuming foods together with family and friends. The traditional Mediterranean diet was molded by the environment, the flora, as well as by poverty and need [4]. At the time when Ancel Keys first described it, the production of food was unindustrialized including foods that nature provided and that were produced with minimal modifications involving a noticeable physical effort, which probably contributed to the multiple benefits derived from combining dietary with lifestyle factors.

Thus, the Mediterranean dietary model has been followed since ancient times by the populations living in the Mediterranean basin, where olive trees and vineyards have been native crops for millennia. The classical Mediterranean triad of olive oil, wine, and bread was combined with legumes and goat milk cheeses. Meat was consumed infrequently on the occasion of religious celebrations such as marriages and festivities of the entire community. This fundamentally plant-based diet was followed by the Greek and Roman populations, which had close ties not only in the ways they ate but also in the forms of government, culture, religion, and philosophy, which were subsequently spread throughout the vast Roman Empire. Between 400 and 800 AD, barbarians invaded the Roman Empire bringing other cultivated plant products as well as meat from game and pigs. The Arabs who arrived in southern Italy in the ninth century brought dried pasta among other products and new forms of gastronomy rich in various condiments and seasonings. With the discovery of America in the fifteenth century other new elements were added to the blend of the Mediterranean diet, such as turkey, tomatoes, beans, corn, potatoes, strawberries, pineapples, coconuts, coffee, and chocolate, conforming the variety of combinations in the Mediterranean dietary model as we know it today. For further detailed information on the history of the Mediterranean diet, it is recommended to consult the review article by Antonio Capurso [7]. In addition to nutritional elements, many other components of the Mediterranean lifestyle came from other traditions, for example the “day of rest”, came from the Judeo-Christian tradition (first mention in Genesis 20:8–11).

## The Mediterranean basin, major religions, and specific dietary practices

**Fig. 3:**
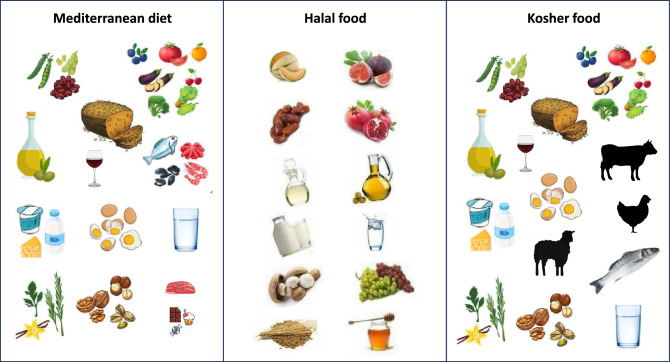
Examples of food components of the Mediterranean dietary pattern (vegetables, fruits, olive oil, whole cereals, legumes, nuts, fish, low amounts of meat and meat products, wine in moderation if not contradictory to religious and social norms or health conditions), foods allowed according to Halal (melon, figs, dates, pomegranate, vinegar, olive oil, milk, water, mushroom, grapes, barley, honey), and Kosher (vegetables, fruits, olive oil, whole cereals, legumes, nuts, fish with fins and removable scales, meat and fowl ritually slaughtered in a kosher manner, wine in moderation if not contradictory to religious and social norms or health conditions) principles. As shown, there are similarities in the foods included in these three eating models

The Mediterranean region was the cradle of the monotheistic religions: Judaism, Christianity, and Islam. Tables 2 and 3 show examples of reference to Mediterranean diet and lifestyle components in the Bible and in the Holy Quran. Indeed, food may shape human relationships and understanding of the world within a cultural and spiritual context. Particularly in occasion of feasts and fasts, religious practices often entail the use of specific foods with important symbolic value. Likewise, there are some types of food that are banned although they are available, and religious codes often exclude whole categories of foods from consumption. This is the case of the main religions that originated in the Mediterranean Sea basin with encouraged/prohibited foods, some of which are part of the Mediterranean diet as we know it today. Figure 3 shows examples of food components of the Mediterranean dietary pattern, as well as foods allowed according to Halal and Kosher precepts.

**Table 2:** Examples of passages from the Bible in reference to Mediterranean food and lifestyle diet components

Components	Passage	Citation
Fruits, vegetables, legumes & whole grains	“Then Jacob gave Esau some bread and some lentil stew. He ate and drank, and then got up and left.”	Genesis 25:34
	“Then their father Israel said to them, If so, do this: take in your bags the choicest things of this land, and bring to that man a gift: a little balm, a little honey, some aromas and myrrh, pistachios and almonds.”	Genesis 43:11
	“We remember the fish we ate in Egypt at no cost—also the cucumbers, melons, leeks, onions and garlic.”	Numbers 11:5
	“The next day Moses entered the tent and saw that Aaron’s staff, which represented the tribe of Levi, had not only sprouted but had budded, blossomed and produced almonds.”	Numbers 17:8
	Seven species – “a land with wheat and barley, vines and fig trees, pomegranates, olive oil and honey.”	Deuteronomy 8:8
	“…brought bedding and bowls and articles of pottery. They also brought wheat and barley, flour and roasted grain, beans and lentils.”	2 Samuel 17:28
	“Then he gave a loaf of bread, a cake of dates and a cake of raisins to each person in the whole crowd of Israelites, both men and women. And all the people went to their homes.”	2 Samuel 6:19
	“Sustain me with raisins; refresh me with apples, for I am sick with love.”	Song of Solomon 2:5
	“The Lord showed me two baskets of figs placed in front of the temple of the Lord. One basket had very good figs, like those that ripen early; the other basket had very bad figs, so bad they could not be eaten.”	Jeremiah 24:1–3
	“At that time Jesus went through the grainfields on the Sabbath. His disciples were hungry and began to pick some heads of grain and eat them.”	Matthew 12:1
	“The kingdom of heaven is like a mustard seed, which a man took and planted in his field.”	Matthew 13:31
Olive oil and olives	“On top of these he placed a thin cake of bread made without yeast, a cake of bread mixed with olive oil, and a wafer spread with olive oil. All these were taken from the basket of bread made without yeast that was placed in the LORD’s presence.”	Leviticus 8:26
	“Together with a grain offering of a tenth of an ephah of the finest flour mixed with a quarter of a hin of oil from pressed olives.”	Numbers 28:5
	“…a land with wheat and barley, vines and fig trees, pomegranates, olive oil and honey.”	Deuteronomy 8:8
	“Then I will send rain on your land in its season, both autumn and spring rains, so that you may gather in your grain, new wine and olive oil.”	Deuteronomy 11:14
	“I will give your servants, the woodsmen who cut the timber, twenty thousand cors of ground wheat, twenty thousand cors of barley, twenty thousand baths of wine and twenty thousand baths of olive oil.”	2 Chronicles 2:10
	“’Yet some gleanings will remain, as when an olive tree is beaten, leaving two or three olives on the topmost branches, four or five on the fruitful boughs’, declares the Lord, the God of Israel.”	Isaiah 17:6
Milk & dairy	“Go up to the land flowing with milk and honey.”	Exodus 33:3
	“He asked for water, and she gave him milk; in a bowl fit for nobles she brought him curdled milk.”	Judges 5:25
	“…honey and curds, sheep, and cheese from cows’ milk for David and his people to eat.”	2 Samuel 17:29
	“He will be eating curds and honey when he knows enough to reject the wrong and choose the right.”	Isaiah 7:15
Fish & seafood	“Then he took the seven loaves and the fish, and when he had given thanks, he broke them and gave them to the disciples, and they in turn to the people.”	Matthew 15:36
	“Jesus said to them, “Come and have breakfast.” None of the disciples dared ask him, “Who are you?” They knew it was the Lord. Jesus came, took the bread and gave it to them, and did the same with the fish.”	John 21:11–13
Physical activity	“A wise man is full of strength, and a man of knowledge enhances his might.”	Proverbs 24:5
	“But they who wait for the Lord shall renew their strength; they shall mount up with wings like eagles; they shall run and not be weary; they shall walk and not faint”	Isaiah 40:31
	“For while bodily training is of some value, godliness is of value in every way, as it holds promise for the present life and also for the life to come.”	1 Timothy 4:8
Moderation	“If you have found honey, eat only enough for you, lest you have your fill of it and vomit it.”	Proverbs 25:16
Meat	“It is not what goes into the mouth that defiles a person, but what comes out of the mouth; this defiles a person.”	Matthew 15: 11
	“I know and I am persuaded in the Lord Jesus that nothing is unclean in itself, but it is unclean for anyone who thinks it unclean.”	Romans 14: 14
	“Eat anything sold in the meat market without raising questions of conscience, for the earth is the Lord’s, and everything in it.”	1 Corinthians 10:25–26

**Table 3:** Examples of passages from the Holy Quran in reference to Mediterranean food and lifestyle diet components

Components	Passage	Citation
Fruits, Vegetables, legumes & Whole Grains	“And when you said, O Moses! We cannot endure one kind of food. Therefore, pray to your Lord to produce for us what the earth grows, its herbs, its cucumbers, its garlic, lentils, and onions”	Surat Al-Baqara (*The* *Cow*) 2:61
	“Would any of you wish to have a garden full of date palms and grapes through which rivers flow underneath? He would have all sorts of fruits in it”	Surat Al-Baqara (The Cow) 2:266
	“And it is He Who produced gardens, both trellised and untrellised, and date palms, and crops of different shape and taste (their fruits and their seeds) and olives, and pomegranates, similar (in kind) and different (in taste). Eat of the fruits when they ripen.”	Surat Al-An’am (*The* *Livestock*) 6:141
	“And shake the trunk of date-palm towards you; it will let fall fresh ripe dates upon you. So eat, drink and be content.”	Surat Maryam (*Mary*) 19:25–26
	“And from it (the earth) we produced whole grains, so that they eat thereof.”	Surat Ya-Sin (*Y.S.*) 36:33
	And remember ye said: “O Moses! We cannot endure one kind of food (always); so beseech thy Lord for us to produce for us of what the earth growth, -its pot-herbs, and cucumbers, Its garlic (Wheat), lentils, and onions.” He said: “Will ye exchange the better for the worse? Go ye down to any town, and ye shall find what ye want!”	Surat Al-Baqara 61
Olive Oil	“And [We brought forth] an olive tree issuing from Mount Sinai which produces oil and [it is a] relish for those who eat.”	Surat Al-Mu’minun (*The Believers*) 23:20
	“The Lord is the Light of the heavens and the earth. The example of His light is like a niche within it a lamp, the lamp is within glass, the glass as if it were a brilliant star lit from [the oil of] a blessed tree, an olive, neither of the east nor of the west, whose oil would almost glow even if untouched by fire. Light upon light. God guides to His light whom He wills. And God presents examples for the people, and God is Knowing of all things.”	Surat An-Nur (*The* *Light*) 24:35
	“With it (the rain) He brings up for you the crops, olives, dates, the grapes and every kind of fruit.”	Surat An-Nahl (The Honey Bees) 16:11
Fish & Seafood	“Lawful to you is game from the sea and its food as provision for the benefit of yourself and those who travel, but forbidden to you is land-game while you are in the state of pilgrimage. And be conscious of God, unto whom you shall be gathered back.”	Surat Al-Ma’idah (*The Table*) 5:96
	“And He it is Who has subjected the sea (to you), that you eat thereof fresh fish.”	Surat An-Nahl (*The* *Honey Bees*) 16:l4
Milk & Dairy	“And Verily! In the cattle, you have a worthy lesson. We give you to drink of that which is in their bellies, between the cud and blood: pure refreshing milk for those who drink it.”	Surat An-Nahl (*The* *Honey Bees*) l6:66
	“(Here is) a Parable of the Garden which the pious are promised, in it are rivers of water the taste and smell of which are not changed; rivers of milk of which the taste never changes; rivers of wine delicious to those who drink, and rivers of clarified honey. They will have, in it, all sorts of fruits; and Grace from their Lord.”	Surat Muhammed or Surat Al-Qital (The Fighting) 47:15
Physical Activity	“Remember Our servant, Job, when he called on his Lord: Satan has afflicted me with exhaustion (by ruining my health) and suffering. For the Lord replied: Strike the ground with your feet! And this is a cool spring of water to wash in and for drinking too.”	Surat Sad (*The letter* *S.*) 38:41–42
Moderation	“Eat and drink, yet not in excess, for the Lord loves not those who commit excess.”	Surat Al-A’raf (The Heights) 7:31
Meat	“He has only forbidden to you dead animals, blood, the flesh of swine, and that which has been dedicated to other than Allah. But whoever is forced (by necessity), neither desiring (it) nor transgressing (its limit) – then indeed, Allah is Forgiving and Merciful”	Surat Al-Nahl (The Bees) 16:115

The food rituals mark essential points of the Mediterranean lifestyle, whatever the religion, giving rise to rites and celebrations in which food is fundamental. During different periods of the year there are abstinence rites, fasting and penitence (i.e., Lent, Shabbat, and Ramadan) indicating the choice of some foods and avoiding the use of others [8]. Likewise, specific festivities and celebrations for each of the religions are commemorated with special dishes. For example, during Eid al-Fitr at the end of the Holy month of Ramadan in the Islamic calendar, a wide variety of particular desserts, bakery items, candy, treats, sweets and cookies are consumed; during Eid al-Adha a spicy lamb is consumed honoring the willingness of Ibrahim to sacrifice his son Ismail as an act of obedience to God's command. Christmas and Easter dishes (roast lamb and other sweets and specific dishes in different regions) as well as the communion of wine and wafers are symbolic foods linked to Christian traditions. In Judaism Passover and Rosh Hashana (New Year) are also celebrated with special tastes—apple and honey are eaten as signs for a sweet year [9]. Likewise, each period of life has its particular kind of food and cooking. Food and its preparation have a deep symbolic meaning [10] that bring together ancient history, beliefs and practices of the community that goes beyond religion [2, 7].

### Judaism

In Judaism, Kashrut is the set of laws defining appropriate foods (in English, it’s called Kosher) [9], which were handed down from their great prophet, Moses in the thirteenth century BC, are outlined in the Torah, and may impact food choices. Other, more subtle, spiritual rules also apply. Traditional Jewish teachings believe that the body is a gift for which the person is responsible; and on a very practical level, an early book of Jewish ethics writes, “It is not possible to understand and become wise in Torah and mitzvot when you are hungry or sickly or when one of your limbs hurts” (Hilchot Deot, Maimonides 3:3). Kosher dietary practices include three principal rules: only to eat animals that have a cloven hoof and chew the cud and are killed in a humane way; only to eat fish with fins and scales (excludes other sea food such as oysters, octopus and squid etc.); and to separate eating milk with meat by a period of 1–6 h according to different traditions [9]. Following these rules may influence how individuals adhere to the Mediterranean diet. Contrarywise, among the abstinence rites, Jewish Sabbath (day or rest) refers to abstinence from work, not from eating since on the Sabbath it is commended to eat at least three full meals. Analysis of the biblical seven species, together with other indigenous foods from the Middle East, have shown to be now scientifically recognized as healthy foods, helping, at least in part, to explain the health benefits of the Mediterranean diet [2].

### Christianity

Many countries in the Mediterranean region have a significant Christian population. While Christianity does not generally have strict dietary commandments, in predominantly Christian regions around the basin there may be specific religious practices during religious seasons that influence dietary habits. For example, during certain periods of the Christian liturgical calendar, such as Lent, some individuals may choose to abstain from certain foods avoiding or reducing meat consumption and adopting a more plant-based diet [9].

One type of Christianity, Adventist Christians, deserves special mention regarding their eating habits and lifestyle because they have been the subject of extensive research for several decades [11–13]. Some studies have shown substantially lower rates of all-cause mortality and cancer incidence among Adventists attributable to the effects of lifestyle and perhaps particularly diet on the etiology of health problems [12]. Likewise, there is evidence of health benefits of the vegan/vegetarian diet of Adventist Christians with higher intake of whole grains, legumes, nuts, and seeds, together with reduced carbon footprints underscoring the adoption of vegetarian diets to address global food supply and environmental sustainability [13].

### Islam

In predominantly Muslim regions around the Mediterranean, adherence to Islamic dietary laws (Halal) may influence food choices. For example, Muslims are prohibited from consuming pork, and they must ensure that meat is slaughtered in accordance with Islamic principles. In Islam, dietary laws are outlined in the Holy Quran, and adherence to these laws is an essential aspect of their faith [9]. These dietary restrictions may shape the choices within the broader framework of the Mediterranean diet. By Islamic law, Muslims are prohibited from drinking alcoholic beverages or foods that include alcohol in its ingredients. This is one of the clear differences between the Mediterranean diet and Middle Eastern Food, as wine is an essential part to the cuisines of the Greeks and other Mediterranean cultures [7]. Alcohol (kham’r), which broadly includes its products such as wine and other intoxicating products has been mentioned in several verses in the holy Quran. Kham’r, according to hadith, covers the ‘intellect’ and hence affects the ability to make rightful decisions (such as attending prayers) and remain conscious. The Quran did reveal that while alcohol has some benefits, the negative effects outweigh the good: they ask you ˹O Prophet˺ about intoxicants and gambling. Say, “There is great evil in both, as well as some benefit for people—but the evil outweighs the benefit.” (Quran, Al-Baqarah, 2:219).

There is a high incidence of lactose intolerance among the people of the Middle East [14], and this may help explain why they rarely drink fresh milk.

Muslims also fast for 12 h while the sun is visible during the holy month of Ramadan and only eat food at night. This precept is worthy of mention in terms of health outcomes. A review article on the effects of Ramadan on health outcomes found that among 53 published articles the most common topic covered was diabetes mellitus followed by metabolic disorders and nutrition [15]. A recent review on the literature regarding Ramadan effects [16] reported that several meta-analyses have shown weight loss following Ramadan fasting. However, full weight regain is often observed 2 to 5 weeks after Ramadan. Other meta-analyses found inconsistencies regarding modifications of lipid profiles and minimal improvements in fasting glucose with inconsistent results regarding insulin. All reviews and meta-analyses suggested sex-specific effects of Ramadan fasting, but again, there were highly heterogeneous results. The authors concluded that there is still need of further studies with robust techniques, such as the hyperinsulinemic euglycemic clamp, to better clarify the effect of Ramadan fasting on metabolic health [16]. Indeed, Ramadan fasting is an important religious issue that needs more attention from a health viewpoint.

### Plant-based patterns in other religions and spiritual practices

This section concerns religions that do not traditionally follow the Mediterranean diet but generally consume plant-based diets that may be similar to the Mediterranean diet.

*Hinduism:* it has diverse dietary practices influenced by cultural, regional, and sect-specific beliefs. Some Hindus follow a vegetarian diet due to principles of non-violence (ahimsa), while others may consume meat but avoid beef. According to Yoga Science and Ayurvedic Medicine, *gunas* (one’s quality or tendency) are classified into three types: *rajas*, *tamas* and *sattwa*; each with its own characteristics. In any person, though all the three are found, one of them is dominant. The type of food one takes influences the dominant *guna*. Yoga Science enumerates the food items that promote a particular *guna*. Thus, these dietary choices may have implications for understanding a person’s psychological functioning [17].

Singh et al., proposed the combination of the Mediterranean diet with the Indian diet showing in a trial that it provided cardiovascular benefits [18]. The Indo-Mediterranean diet is rich in vegetables and fruits, nuts, whole grains, beans, and additionally has a high content of particularly spices (e.g., turmeric, coriander, cumin, cloves, cardamom), which may have robust anti-inflammatory and antioxidant properties [19]. However, after the initial trial, there is lack of further studies confirming these potential effects.

*Buddhism:* monks often follow a vegetarian diet as part of their monastic discipline, reflecting principles of compassion and non-harming. However, dietary practices can vary among different Buddhist traditions. Vegetarian diets have shown beneficial effects both for human health and for the sustainability of the planet due to lower greenhouse gas emissions compared to diets that include animal foods [20]. A meta-analysis on the beneficial effects of vegetarian and vegan diets on health outcomes found a significant protective effect of a vegetarian diet on the incidence and/or mortality from ischemic heart disease and incident total cancer. Vegan diet conferred a significant reduced risk of incident total cancer [21].

*Sikhism:* this religion does not have strict dietary laws, but there are guidelines for maintaining a simple and balanced diet. Sikhs are encouraged to avoid intoxicants, including tobacco and alcohol [9].

*Shinto:* this religion deserves a special mention since about half of Japanese people follow it.

Japan is the country with the longest life expectancy in the world, the largest number of centenarians, and the fastest increase of aging in the world [22]. A higher adherence to the Japanese diet was associated with a reduced incident cardiovascular disease and mortality as well as a lower incidence of stroke [23]. There is also evidence showing an inverse association of a higher adherence to the Japanese diet with a lower risk of disability and incident Alzheimer’s disease [24].

The foundation of the "Shinto" religion is the unique custom to respect and appreciate what nature presents to humans by discovering deities in nature to be praised in many aspects of daily life or at special ceremonies and festivals with food offerings to deities throughout the seasons. The offering meals (*shinsen*) consist of traditional staple foods such as rice, mochi (rice cake), sake, salt, and water, along with the best catch and harvest available in the season from the mountains to the sea. Shinto priests conduct the purification ritual by washing their entire bodies prior to cooking the offering meals. Such practices were registered within the UNESCO Intangible Cultural Heritage in December 2013 under the title of "*Washoku*, traditional dietary cultures of the Japanese" [25].

Interestingly, there is literature comparing the Mediterranean to the Japanese diet, given their impact on longevity. The main similarity between these healthy diets is the substantial consumption of vegetables, legumes, and fruit. Fish is consumed regularly, more in Japan than in Mediterranean countries, while red meat is consumed more sparingly. The majority of fat consumed in Japan is rice bran oil, whereas in Mediterranean countries, it is olive oil. In addition, the Japanese diet contains abundant fermented foods, and seaweed. Thus, it is plausible to combine the two models to achieve further health benefits [25]. Both dietary patterns are plant-based, minimally processed, and sustainable for their respective regions and have been associated with significant longevity. Additionally, both dietary patterns are part of broader healthy lifestyle including abstaining from smoking, engaging in regular physical activity, having low-stress levels, and a sense of community, spirituality/ religiousness, and purpose [26]. A recent review aiming to identify correlations between healthy longevity and both the Mediterranean and the Japanese diet identified 10 studies concluding that in all of them, very old participants reported good adherence to both Mediterranean and Japanese eating behaviors, acceptable anthropometric characteristics, active social life, and regular physical activity [27].

## Intersection points between the Mediterranean diet and spirituality/religion

**Fig. 4:**
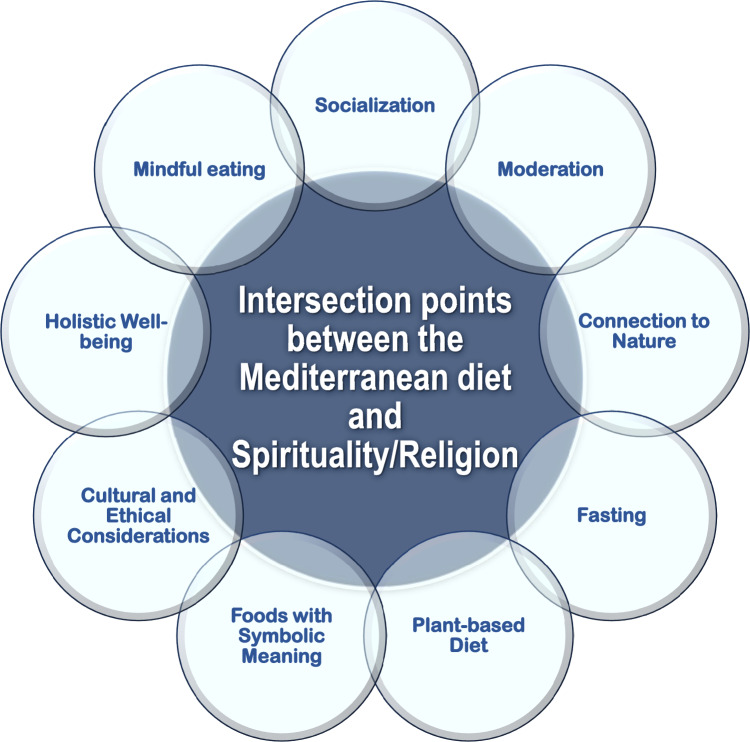
The aspects by which Mediterranean diet and spirituality/religion are interconnected are shown in the graph

The Mediterranean diet and spirituality/religion can be interconnected in various ways, as both involve elements of well-being, mindfulness, and connection to certain values. People can make their dietary choices based on spiritual beliefs, ethical considerations, symbolic and spiritual reflections, and a desire for overall well-being. Therefore, spirituality/religion can influence dietary patterns, and in the context of the Mediterranean region, where various religions are practiced, there can be some connections between religious beliefs and the dietary choices that align with the Mediterranean diet. In the following subsections we describe some aspects where the Mediterranean diet and spirituality/religion may align (Fig. 4).

### Mindfulness and mindful eating

Many spiritual practices encourage mindfulness, presence, and gratitude in daily activities. Practicing mindful eating, where individuals focus on the sensory experience of eating, can be a spiritual practice. The Mediterranean diet emphasizes mindful and intentional eating by savoring and appreciating the flavors of fresh, whole foods, which can align with the concept of being aware and present during meals. A recent study showed that children with higher mindful eating had higher environmental beliefs and higher scores of adherence to the Mediterranean diet, while these scores were lower in obese children [28]. Furthermore, empirical literature suggests that some practitioners of mindfulness, with origin in Buddhist traditions, exhibit indicators of enhanced functioning including pre-eminent physical health and resistance to disease, healthy aging and improved cognitive processing, greater resilience and fearlessness, more self-less and pro-social behaviors, some control over normally autonomic responses, and possibly some paranormal functionality. Such enhancements in normal human functioning might want to consider adopting this practice in order to develop great capability [29]. However, there are insufficient studies of expert meditators and further research is needed to explore the relationship between levels of attentional skill and increases in functionality.

### Vegetarianism and veganism

The Mediterranean dietary pattern is not a strict vegetarian diet but most of its components are of plant origin. Some spiritual practices advocate for compassion and non-harming, which can lead individuals to adopt vegetarian or vegan diets. These choices are often based on ethical considerations related to the treatment of animals and the desire to minimize harm [30]. The EAT-Lancet Commission quantitively describes a universal healthy reference diet, based on an increase in consumption of healthy foods, most of plant origin (i.e., vegetables, fruits, whole grains, legumes, and nuts), and a decrease in consumption of unhealthy foods (i.e., red meat, sugar, and refined grains) that would provide major health benefits, and also increase the likelihood of attainment of the sustainable development goals [31]. Likewise, a recent comprehensive review of the literature suggested that plant-based diets with moderate lipid content, characterized by consumption of vegetables, fruits, whole grains, legumes, nuts, and unsaturated fats, with low to moderate amounts of poultry and seafood, and low amounts of red meat and sugar can offer substantial health benefits [32], which is in line with the Mediterranean diet principles.

### Fasting and cleansing

Fasting is a common practice in many religions and spiritual traditions as a means of purification, self-discipline, and spiritual growth. Some individuals incorporate periodic fasting or cleansing diets into their spiritual practices. During Ramadan, the ninth month of the Islamic calendar, Muslims worldwide observe a month of fasting as a commemoration of Muhammad's first revelation, and regarded as one of the Five Pillars of Islam. Lent fasting is the solemn Christian religious observance in the liturgical year commemorating the 40 days Jesus spent fasting in the desert and enduring temptation by Satan before beginning his public ministry [33]. Jewish Fasts comprise: 10 Tevet, 17 Tammuz, and 9th Av in memory of the destruction of the two Temples in 586 BCE and 70 CE; Yom Kippur 24 h fast for Repentance. There are also additional fasts for the extremely observant – before each new moon and three day-long fasts after the major 8-day festivals of Passover and Sukkot (Tabernacles) to counter the effects of over-eating during these periods [9]. Traditionally, Buddhist monks follow eight precepts, one of which specifies that one must not eat after the noon meal. This is not considered a kind of fasting, but a simple and moderate way of eating which is said to aid one's meditation and health. Fasting for some days (along with other ascetic practices like bathing in freezing water) is considered to be highly effective at producing ascetic spiritual power as well as having cleansing properties and producing mental clarity [34].

Interestingly, fasting has gained significant attention in recent years for its potential health benefits in various body systems. Fasting has been related to better cardiovascular health (blood pressure, cholesterol and triglycerides profiles), as well as with enhancing insulin sensitivity, promotion of weight loss, and prevention and treatment of type 2 diabetes (T2D). Fasting has been also related to improving immune function, reducing inflammation, enhancing autophagy with supporting beneficial effects against infections, cancer, and autoimmune diseases. Other benefits associated with fasting relate to its neuroprotective and longevity promoting properties [35]. Diverse types of fasting have been studied but the one with the largest body of evidence in the literature is intermittent fasting (IF). Most studied forms of IF include alternate-day fasting, modified alternate-day fasting, and time-restricted eating. A review on observational or interventional studies of IF in humans published between January 2000 and June 2021 showed that the different types of IF had significant benefits on body composition, inducing weight loss, and reducing fat mass. However, modification in cardiometabolic parameters show more divergent results with high heterogeneity in study designs, small sample sizes, and short-term interventions [36]. A meta-analysis investigating the effects of IF vs. a control diet reported that IF was effective for weight loss and specific cardiometabolic health markers in persons with prediabetes or T2D. Additionally, IF was associated with a reduction in body weight and body mass index (BMI) compared to caloric restriction, without effects on glycemic markers, lipid profiles or blood pressure [37].

In reference to religious fasting, a review among 17 clinical studies exploring the potential beneficial impact of the dietary pattern of Christian Orthodox fasting on human health showed favorable effects concerning glucose and lipid control, with inconclusive data for blood pressure. There were no dietary deficiencies for iron and folate but deficiencies for calcium, vitamin B2, and vitamin D were recorded in Orthodox monks [38].

A systematic review and meta-analysis on the advantages of different IF protocols for metabolic homeostasis in participants with obesity, T2D, and metabolic syndrome using data from 64 reports with sufficient quality and 47 for quantitative analyses showed that alternate-day fasting protocols promoted the major beneficial effects in the improvement of dysregulated metabolic conditions vs. time-restricted fasting and religious fasting protocols. Participants with obesity and metabolic syndrome were the most benefited with the introduction of these interventions, through the improvement of adiposity, lipid homeostasis and blood pressure. For participants with T2D, the impact of IF was more limited; thus, IF seems to differently impact metabolic homeostasis depending on an individual's basal health status and type of metabolic disease [39].

Several mechanisms have been proposed to help explain the beneficial health effects of fasting, including enhancing parasympathetic activity (mediated by the neurotransmitter acetylcholine) in the autonomic neurons that innervate the gut, heart, and arteries, resulting in improved gut motility and reduced heart rate and blood pressure. In addition, by depleting glycogen from liver cells fasting results in lipolysis and the generation of ketone bodies, causing a reduction in body fat; IF enhances insulin sensitivity of muscle and liver cells and reduces insulin-like growth factor-1 production. Levels of oxidative stress and inflammation are reduced throughout the body and brain in response to IF [40].

### Connection to nature

Many spiritual traditions emphasize a connection to nature and the environment. The Mediterranean diet includes locally sourced, seasonal, and plant-based foods, reflecting a harmonious relationship with the natural world. It can be seen as a way of aligning with nature and promoting environmental sustainability with an evolution from a healthy dietary pattern to a sustainable dietary pattern, in which nutrition, food, cultures, people, environment, and sustainability all interact into a new model of a sustainable diet [4].

According to the EAT-Lancet commission, a global transformation of the food system is urgently needed. The transformation to healthy diets by 2050 will require substantial dietary shifts, including a greater than fifty percent reduction in global consumption of unhealthy foods, such as red meat and sugar, and a greater than one hundred percent increase in consumption of plant-based healthy foods, such as nuts, fruits, vegetables, and legumes [31].

### Moderation

Spirituality in various traditions often emphasizes the value of moderation and balance and promotes self-control in various aspects of life. The Mediterranean diet emphasizes a balanced intake of fruits, vegetables, whole grains, lean proteins, and healthy fats, and encourages moderate consumption of these healthy choices, reflecting the principles of temperance. Interestingly, at the time of the Seven Countries Study, Cretan farmers were upset telling the American interviewers how poor was their diet with so little meat, ignoring that probably that was one of the main explanations for their outstanding health, in combination with their heavy work in the fields [1]. Reasonably, the notion that excess food has unhealthy consequences is not considered in times of scarcity, when increased weight may be perceived as good health. At that time, people would hardly believe a physician’s advice on the benefits of a frugal diet, composed of unexpensive products that were considered foods for the poor and a low intake of meat and sweets, in combination with constant physical effort. This type of diet and lifestyle, which in years to come would be recognized as one of the healthiest in the world, was the result of consuming available food at the limit of subsistence, where a sparing diet composed of products provided by nature was a necessity more than a life choice. This may help to explain why once exposed to the economic boom after the Second World War, these societies tended to abandon this way of eating and living preferring the indulgences of the Western model of diet and lifestyle [4]. The difficulties in transmitting messages of prevention today is similar to these historical testimonies. It is challenging to convince the lay public that they should eat sparingly and move constantly when they eagerly search for an indulgent lifestyle.

### Holistic well-being

Many spiritual traditions emphasize overall well-being, not just physical health. The Mediterranean diet, with its focus on a variety of nutrient-dense foods, is often associated with promoting not only physical health but also mental and emotional well-being [41, 42]. Comprising not only nutrition but also many components not directly linked to its nutrient content (Table 1), it can be seen as a way of supporting holistic well-being. This can represent a better quality of life for those more adherent to the Mediterranean diet, which has been reported in patients with depression and other mental conditions [42, 43], T2D [44], and even people during COVID-19 lockdown [45], among others.

### Social and communal aspects

One of the most prominent cultural values promoted in the Mediterranean diet and lifestyle model is the idea of conviviality and the pleasure of shared meals (Table 1). Sharing meals is a common practice in many spiritual traditions, emphasizing the importance of communal experiences. The Mediterranean diet, with its focus on preparing and consuming meals in the company of other people, as well as social gatherings, aligns with the idea of building connections through shared meals [46].

Regarding the origins of the Mediterranean Diet, Berry et al. compared how foods were consumed in ancient times respect to current practices [2]. They discuss how present trends of eating while watching television promote unhealthy, quick meals and exclude social/family communication. Conversely, in biblical times, mealtimes nurtured relationships and was an opportunity for communication. For example, the Last Supper of Christianity was the traditional Passover Seder meal in Judaism, and they were styled on the Greek symposium in which people reclined and communicated while eating [2].

### Cultural and ethical considerations

Some spiritual practices involve specific dietary rituals or traditions that may influence ethical and cultural choices. The Mediterranean diet, rooted in the cultural practices of the region, can resonate with individuals seeking alignment with certain cultural or ethical values. Shared meals and feasts may hold cultural or religious significance, fostering a sense of community and shared experience.

Alongside the popularity of the Mediterranean diet, which continues to spread throughout the world, adherence to this remarkably healthy dietary pattern is being abandoned in countries located in the Mediterranean region where the first beneficial effects were reported [47–49]. Several reason can help explain this phenomenon, mostly related to the influence of the Western economy, to the globalization of food production and consumption, as well as to the rising urbanization, technology, and tourism. Factors contributing to this hazard include the propagation of fast-food and sugar-sweetened drinks availability and consumption and the economic crisis, which force underprivileged populations to choose low-cost industrial food, full of empty calories but poor in nutrients [50]. Such aspects signify widespread dangers for the conservation and transmission of the intangible Mediterranean diet heritage for the forthcoming generations.

Therefore, it is vital that governments and the society commit to develop programs aiming to preserve the invaluable heritage of the Mediterranean diet based on cultural and traditional foundations, encouraging the communities towards sustainable diets and food diversity, which offer short and long-term health outcomes as well as environmental benefits.

### Foods with symbolic meaning

As discussed before and illustrated in Tables 2 and 3, certain foods may have symbolic importance in spiritual practices and religions originated in the Mediterranean region. For example, grains, fruits, and other natural foods may symbolize abundance, purity, fertility or spiritual significance. For all societies, and therefore everyone, food has an emotional quality and carries symbolic meanings [51]; it is difficult to forget the foods someone has grown up with and the type of foods served in the social and family context. Eating habits are both symbols in themselves and incorporate symbols; they are the permanent determinations that the person gives him/herself to be nourished, which later adhere to community life and shape the food culture [52]. Food choices imply a complex mechanism of interaction between biological, social, and cultural processes, including food preferences and aversions, values, symbolism and traditions, together with the organoleptic characteristics of food [52].

The alienation of human beings in modern societies is reflected in the loss of the symbolic value of food. In the industrial societies, food is just food. In addition, globalization has favored the socio-cultural-food habits to become uniform in industrial societies, adopting unhealthy diets and abandoning the healthier traditional ways of eating and living [53, 54], as it happens with the Mediterranean diet [48, 50].

## Perspective

The Mediterranean dietary model is not only an optimal combination of nutritional elements, but it is also a dietary pattern that culturally and historically goes together with other lifestyle healthy factors, such as social engagement, physical exercise, palatable eating, and adequate rest, respecting traditions that have been transmitted from generation to generation for a long historical time. Furthermore, research evaluating the impact on the environment associated with food patterns have concluded that a shift from animal-based diets towards plant-based diets, such as the Mediterranean diet, are more environmentally friendly, therefore, more sustainable [31, 32].

The various connections between Mediterranean diet and spirituality/religion explored in this article (Fig. 4), may be instruments to stimulate and support the adherence to this model of healthy eating and living, recognizing that healthy choices can be linked, at least in part, to the symbolic value of food, its history and cultural context, as well as the possibility of eating and living consciously in opposition to excess eating, sedentarism, and low quality sleep linked to stress and mental conditions that deregulate the control of hunger and satiety and favor the development of obesity [55], a pandemic of today’s world, especially after COVID-19 pandemic [56, 57]. Indeed, obesity is currently a major public health problem associated with the development of several non-communicable diseases and with all-cause and specific-cause mortality. Persons with obesity are at higher risk for cardiovascular disease, dyslipidemia, fatty liver disease, type 2 diabetes, and several types of cancers [58]. Obesity in children and adolescents is particularly worrying because those affected will most likely be obese for the rest of their lives. The prevalence of child and adolescent obesity has reached remarkably high levels in most high-income countries and is growing in many low-income and middle-income countries [59]. Obesity arises when a combination of genetic and epigenetic factors, behavioral risk patterns and environmental and sociocultural influences affect body weight regulation. The main environmental obesogenic factors are related to build environment (i.e., city plan), transport and school, TV and screen-related immobility, video games, smart-phone and social media access followed by food availability factors such as imbalanced ingredients, pollutants, portion size, sugar-sweetened beverages, and junk foods supported by publicity, among others [60]. Industry and the media, including social media, can have a considerable influence on the food and physical activity behaviors of children [59]. Other social influences, such as the sociotype, must be considered [52]. The sociotype relates to an individual’s dynamic interactions with the surrounding social environment throughout life and comprises three domains: the Individual, Relationships, and Context all of which are affected by nutrition by ensuring the following dimensions of food security: utilization (metabolic fuel and health); accessibility (physical and economic); and availability (the right to nutritious food for all citizens). The sociotype is influenced by multiple factors, including diet–gene interactions, allostasis, microbiota, oxytocin, and culturally through mate selection, family bonds, social communication, political ideologies, and values [52].

Despite several guidelines to reduce body weight and adiposity developed by scientific societies and governments as well as an extensive medical literature on the topic, there are still controversies about the role of dietary fats and carbohydrates on weight gain [61]. Several dietary models with diverse macronutrient composition, including the Mediterranean diet, have been proposed for the management and prevention of obesity with similar results when proposed regimes are energy-restricted. The Mediterranean diet is a plant-based, high-fat, high-unsaturated fat dietary pattern that has been consistently associated with lower rates on non-communicable diseases and total mortality. For these merits this dietary pattern might also be used advantageously for weight loss [61, 62].

Nevertheless, the healthy dietary and lifestyle choices of the Mediterranean diet (i.e., high consumption of fruits, vegetables, whole grains, legumes, nuts, and olive oil, with moderate consumption of fish and poultry and low consumption of red meat and dairy products) are often influenced by the availability of local produce and cultural preferences rather than religious beliefs [63]. While not strictly religious, cultural practices, which are often intertwined with religious beliefs, can influence dietary choices.

It is worth mentioning that reviewing the history and cultural roots of food in the Mediterranean diet means dealing with a complex phenomenon, due to the variations based on local customs, traditions, and the predominant religion in a particular area. Thus, there cannot be a *single unique* Mediterranean diet, but *many* Mediterranean diets. It is as well important to recognize that the connection between spirituality/religion and dietary choices is highly subjective, and people from various spiritual backgrounds may interpret and integrate these concepts differently. Some persons may consciously choose the Mediterranean diet as a way of aligning their dietary and lifestyle habits with their spiritual or philosophical beliefs, while others may not find a direct connection. Ultimately, the relationship between spirituality/religion and diet and lifestyle is a personal and subjective aspect of an individual's lifestyle.

The confirmation that the Mediterranean diet represents a paradigm of healthy eating and lifestyle has aroused great interest worldwide with a growing number of studies that apply the principles of this dietary and lifestyle pattern in epidemiological and clinical investigations as well as in nutritional guidelines outside the Mediterranean region [1, 47–49]. Therefore, the extrapolation of the benefits of the Mediterranean diet in other contexts, with due adaptations to local characteristics, not only of the diet and available foods, but also of culture and traditions is feasible. Further research on the transferability and effectiveness of this healthy eating pattern and lifestyle in non-Mediterranean populations and its health benefits is needed.

## Conclusions

For thousands of years, the Mediterranean basin has been a crossroads of civilizations and religions, which have contributed to the generation of the Mediterranean diet and lifestyle as we conceive it today. This healthy way of eating and living is the result of adoptions, transmission, and dissemination over time, of wide-ranging food practices and local products and foods arriving from abroad (i.e., Asia, India, the Middle East and America), as well as of the development of international exchanges for agricultural products, and the growing mobility of populations.

While there may be some overlap between dietary practices in the Mediterranean region and spiritual/religious customs, the Mediterranean diet itself is not tied to any specific religion. It is more a reflection of the culinary traditions and lifestyle choices of the people in the Mediterranean basin.

The Mediterranean diet and lifestyle consist of choosing healthy foods, in the background of ancient traditions deep-rooted in the historical and cultural characteristics of rural societies that for centuries have followed them without being aware of the immense benefits this implicates. We may say that the Mediterranean diet is a lifestyle in which time is valued, with profound connections and respect to the natural, social, and inner dimensions, as well as an archetype of adaptation, resilience, and evolution. This diet has been associated with great health benefits and it is also tasty, which may help to follow it for a longer time as a usual eating habit and lifestyle.

Overall, both the Mediterranean diet and spirituality share a common ground in promoting a holistic approach to health and well-being. It is also possible that the intensity of the relationship between the Mediterranean diet and religion/spirituality might be affected by how religious/spiritual an individual is. Nevertheless, preventive medicine would benefit from a more holistic approach to nutritional and lifestyle factors and conditions, especially when confronted with great challenges of the spread of unhealthy eating and lifestyle models, so prevalent in the world today.

## Data Availability

No datasets were generated or analysed during the current study.
